# Rehabilitation in Cases of Maxillary Lateral Incisor Agenesis Using Zirconia Implant and Abutment: Finite Element Analysis and Systematic Review

**DOI:** 10.1111/jerd.70080

**Published:** 2025-12-20

**Authors:** Leonardo Folmer Rodrigues da Silva, Ivan Onone Gialain, Marina Guimarães Roscoe, Omar Melendres Ugarte, Paolo Maria Cattaneo, Josete Barbosa Cruz Meira

**Affiliations:** ^1^ Department of Biomaterials and Oral Biology School of Dentistry of University of São Paulo São Paulo Brazil; ^2^ Research Program on Integrated Dental Sciences Faculty of Dentistry of the University of Cuiabá Cuiabá Brazil; ^3^ Faculty of Dentistry, Oral and Craniofacial Sciences King's College London London UK; ^4^ Faculty of Medicine, Dentistry and Health Sciences, Melbourne Dental School The University of Melbourne Parkville Australia

**Keywords:** anterior maxilla, biomechanical evaluation, finite element analysis, maxillary lateral incisor agenesis, zirconia implant

## Abstract

**Objective:**

The use of narrow‐diameter implants has emerged as a strategy to compensate for the reduced bone dimensions of maxillary lateral incisor agenesis (MLIA). This study integrates finite element analysis (FEA) and systematic review (SR) to assess the biomechanical and clinical viability of implant‐supported crowns in MLIA scenarios.

**Materials and Methods:**

Three‐dimensional FEA were built, comprising a control and an atrophic model representative of an MLIA case. Simulations were performed using implants with varying diameters and materials. The risk of implant and abutment mechanical failure and bone resorption were evaluated. The SR was designed to evaluate the consistency between in silico predictions and clinical outcomes.

**Results:**

Reduced implant diameter was associated with an increased risk of implant fracture and bone resorption. All implants and abutments in the simulated models exhibited stress values below the critical threshold for titanium and zirconia failure, indicating a low mechanical failure risk under simulated conditions. Additionally, among the 25 studies included in the SR, 19 reported successful outcomes for implant therapy, but no zirconia implants were identified.

**Conclusions:**

Titanium implants with regular diameter combined with hybrid abutments demonstrated favorable biomechanical behavior and seem a reliable option for MLIA rehabilitation, offering both structural integrity and esthetic benefits.

**Clinical Significance:**

The implant–prosthetic rehabilitation of MLIA has demonstrated high reliability and predictability in both esthetic and functional outcomes, especially when performed through a multidisciplinary approach involving orthodontics, periodontics, implantology, and prosthodontists. Long‐term studies are still necessary to validate the longevity and performance of zirconia implants in MLIA.

## 1 Introduction

Maxillary lateral incisor agenesis (MLIA) is one of the most common forms of dental agenesis in humans, after the third molar and mandibular second premolar [1, 2]. This condition requires particular attention due to its location in the anterior region of maxilla, often referred to as the “esthetic zone” [3]. Dental agenesis in this region compromises smile balance and symmetry, potentially leading to a negative impact on the patient's self‐esteem [4].

Traditionally, orthodontically treatment involving space closure and canine recontouring has been the preferred approach, supported by numerous studies reporting favorable long‐term esthetic outcomes, superior than prosthetic alternatives [5, 6]. In recent years, two systematic reviews have been conducted to determine the most suitable treatment option for MLIA [5, 6], both of which concluded in favor of space closure. Additionally, this approach can be completed without the need to wait for facial growth to cease, which is required before placing an implant in the esthetic zone [7].

At the same time, advances in biomaterials and surgical techniques, particularly in regions with high esthetic demands, have contributed to the growing adoption of dental implants for anterior tooth replacement [8]. Consequently, there is increasing support for shifting MLIA treatment toward orthodontic space opening to placement of an implant‐supported crown in the lateral incisor position [9]. Those who prefer this second approach argue that maintaining the canine guidance results in a more stable and balanced occlusion [6]. Experts also agree that implant‐supported crowns should be prioritized in adult patients, concave or plane facial profile, Class III or Class I malocclusion with a tendency toward Class III, presence of inferior diastemas, deep overbite and large, well‐positioned, and dark‐colored canines [10]. Additionally, achieving smile symmetry is particularly challenging when space closure through canine mesialization is performed in cases of unilateral agenesis [11].

Careful consideration is required when performing oral rehabilitation with implants, and a multidisciplinary team is essential to ensure predictable and successful outcomes. Recent studies have highlighted several periodontal challenges associated with implant insertion in the esthetic zone, including buccal gingival recession, incomplete papilla fill in the interproximal space, long‐term infraocclusion of the prosthetic crown, and, in some cases, peri‐implant bone resorption [12, 13, 14]. These issues are particularly pronounced in cases of agenesis, since the absence of the lateral incisor leads to morphological alterations in the alveolar process, resulting in reduced buccal bone thickness (BBT) [15, 16, 17]. In the atrophic maxilla, some authors have recommended the use of implants with reduced diameters to maximize the available buccal bone thickness (BBT) and enhance esthetic outcomes [10]. However, while an implant with smaller diameter preserves more BBT, it may negatively impact the stress distribution, potentially increasing the risk of peri‐implant bone resorption due to overload [18, 19].

Given these anatomical constraints in MLIA, the choice of implant and abutment material becomes equally critical. Titanium implants and abutments have long been considered the gold standard for implant‐supported crowns [20]. However, the esthetic aspect may be compromised by a grayish discoloration in the peri‐implant mucosa, particularly in patients with a thin gingival biotype on the buccal side [21]. To address this limitation, zirconia has been proposed as a potential alternative to metal due to its superior fracture toughness compared to other ceramics. Recent studies have shown that zirconia implants and abutments demonstrate a comparable survival rate to titanium while providing better esthetic results [22, 23, 24]. However, zirconia presents a higher risk of fracture, which may compromise the long‐term success of the treatment [25], especially because implant fracture represents a critical complication in oral rehabilitation. Assessing the trade‐off between the esthetic needs and mechanical requirements of implants in the esthetic zone is not a simple task.

Finite element analysis (FEA) is a valuable tool to evaluate and predict the implant biomechanical behavior, aiding in the selection of the most suitable design for each treatment scenario, including MLIA cases. With the advancement of zirconia implants, several FEA studies have investigated their biomechanical behavior under different conditions [26, 27, 28, 29, 30]. However, its accuracy in predicting clinical outcomes depends on the ability to provide the correct inputs and to select an output coherent with the failure mechanism of interest for the purpose of the analysis. In addition, the FEA results must be consistent with clinical trials outcomes. Finally, since FEA aims to replicate real clinical conditions in a virtual environment, computerized tomography (CT) imaging should be used to generate patient‐specific jawbone geometries [26].

In this context, this study aimed to predict, through finite element analysis, the risks of marginal bone loss and abutment or implant fracture associated with mechanical overload in MLIA cases, considering variations in implant diameter and material. Additionally, a systematic review was performed to assess the clinical outcomes of MLIA rehabilitation using implant‐supported single crowns and to evaluate the consistency between in silico simulations and clinical findings. The research hypothesis was that using a narrow‐diameter implant and zirconia material would increase the risk of biomechanical failure in implant‐supported crowns for MLIA cases.

## 2 Material and Methods

### 2.1 Finite Element Analysis

Two three‐dimensional models of the anterior region of maxilla, corresponding to the upper lateral incisor region were designed:

- Control model (C): A patient‐based model representing normal maxillary bone dimensions without agenesis;
- Atrophic model (A): A model simulating an atrophic maxilla with bone dimensions characteristics of an MLIA case, based on data from the literature.

For the control model, the study was submitted and approved by the Ethics Committee on Research (protocol number: 77129424.5.0000.0075) for the use of DICOM files from a computerized tomography scan to generate the geometric models. Segmentation of the maxilla was performed using the Blue Sky Plan software (Blue Sky Bio, Libertyville, IL, USA) and the segmented model was subsequently exported to MeshMixer software (Autodesk Inc., San Francisco, CA, USA) to create the tridimensional object. For the agenesis model, the same maxillary geometry was modified in the region of lateral incisor, so that bone dimensions were consistent with the values reported in the literature [17] to simulate an MLIA case.

**FIGURE 1:**
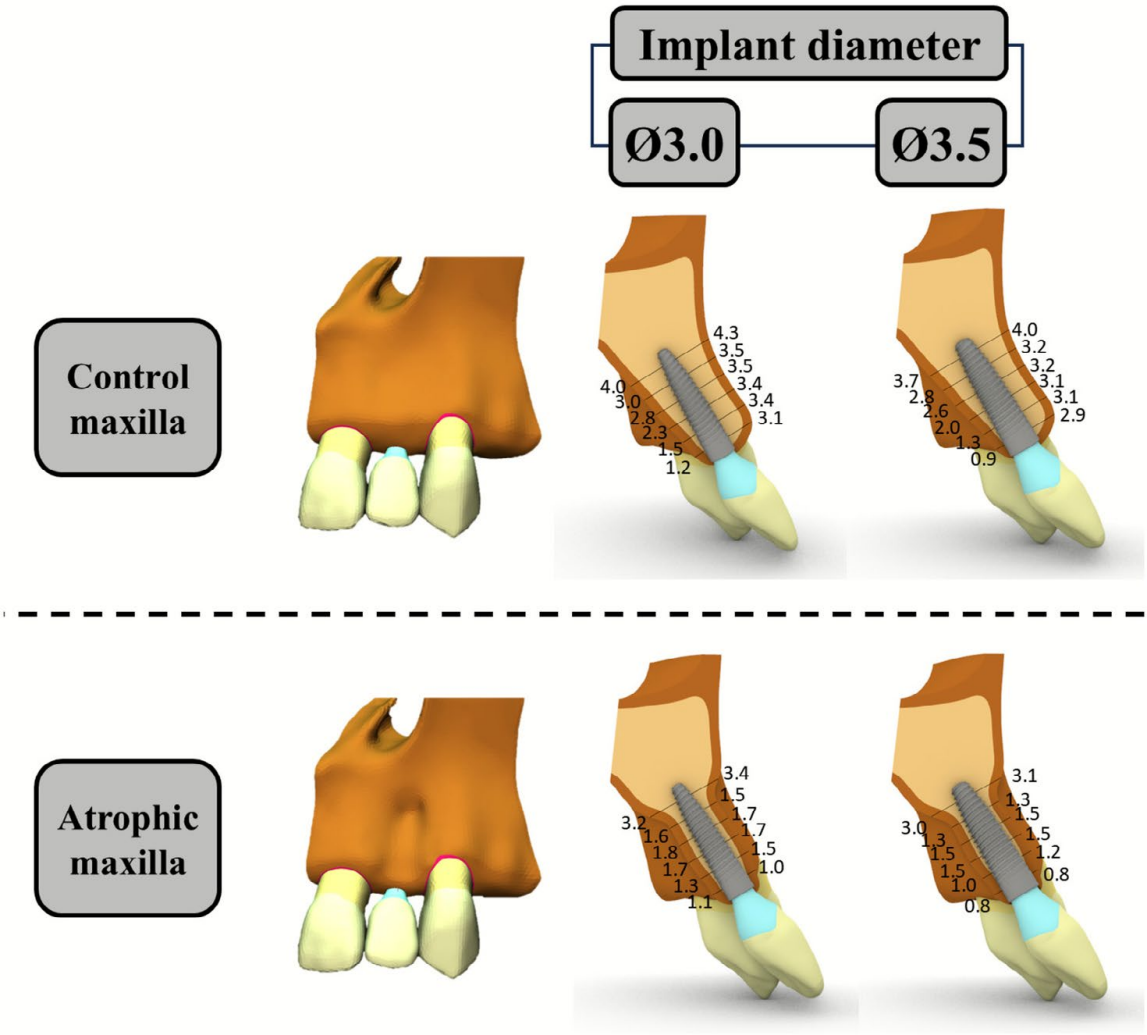
Three‐dimensional representations of the maxilla in both control and atrophic conditions. The left panel illustrates the buccal view, while the right panel presents a sagittal plane section on the upper lateral incisor region in mesial view, presenting the buccal and palatal bone dimensions in mm of the lateral incisor region. Ø: Implant diameter in mm.

Both maxillary geometries were constructed using Rhinoceros program (version 7, Robert McNeel & Associates, Seattle, WA, USA). Implant, abutment and prosthetic crown were added to the model to simulate the rehabilitation of the lateral incisor. The implant diameter varied between 3.0 and 3.5 mm, with a standardized length of 13 mm for all simulations. The prosthetic crown was made of lithium disilicate in all cases. Additionally, the adjacent natural central incisor and canine were included in the model, consisting of enamel, dentin and periodontal ligament. The crowns geometries were imported from the University of Dundee's virtual library (https://sketchfab.com/DundeeDental) and its dimensions were adjusted to standard values available at Wheeler's Dental Anatomy, Physiology and Occlusion [31]. Figure 1 illustrates the simulated geometric models.

For each maxillary model (control and atrophic), different implant systems were simulated, varying implant and abutment materials:

- TT group: Titanium implant and Titanium abutment;
- TH group: Titanium implant and Hybrid abutment, which is composed of a zirconia portion customized over a titanium‐base (ti‐base);
- ZZ group: Zirconia implant and Zirconia abutment.

For each group two implant diameters (3.0 and 3.5 mm) were simulated, except for the ZZ group, which was only simulated with a 3.5 mm implant diameter, since this is the narrowest zirconia implant commercially available for screw‐retained crowns. Table 1 summarizes the total number of FEA models in this study.

**TABLE 1:** Total number of simulated FEA models.

Titanium implant (T)	Titanium abutment (T)	Titanium implant (T)	Hybrid abutment (H)	Zirconia implant (Z)	Zirconia abutment (Z)
TT‐Ø3.5‐C TT‐Ø3.0‐C TT‐Ø3.5‐A TT‐Ø3.0‐A		TH‐Ø3.5‐C TH‐Ø3.0‐C TH‐Ø3.5‐A TH‐Ø3.0‐A		ZZ‐Ø3.5‐C ZZ‐Ø3.5‐A	
Total: 10 models					

Abbreviations: A, atrophic maxilla; C, control maxilla; H, hybrid abutment; T, titanium; Z, zirconia.

The MSC. Apex software (MSC Software, Santa Ana, CA, USA) was used for discretization and assignment of the materials' elastic properties. The element density was determined through a convergence mesh test, following the methodology described in the book chapter “Finite Element Analysis in Dentistry” [32]. The total elements number in the simulated models ranged from 730,456 to 757,892. The materials were represented as linear, elastic, homogeneous, and isotropic. The elastic properties of each material are presented in Table 2.

**TABLE 2:** Elastic properties of simulated materials and critical value for failure.

Material	Young's modulus (GPa)	Poisson's coefficient	Critical value for failure	References
Titanium—Ti‐6Al‐4V grade 5 (implant)	114	0.34	YS: 483 MPa	[33, 34]
Titanium—cpTi grade 4 (abutment and ti‐base)	110	0.30	YS: 795 MPa	[33, 34]
Zirconia (abutment and implant)	220	0.30	UTS: 786 MPa	[35, 36]
Lithium disilicate crown	95	0.25		[37]
Cortical bone	13.7	0.30	4000 μɛ = 109.6 μJ/mm^3^	[38]
Trabecular bone	1.37	0.30	—	[38]
Enamel	80	0.30	—	[39]
Dentin	18.6	0.31	—	[40]
Periodontal ligament	0.15	0.45	—	[41]

Abbreviations: UTS, ultimate tensile strength; YS, yield strength.

**FIGURE 2:**
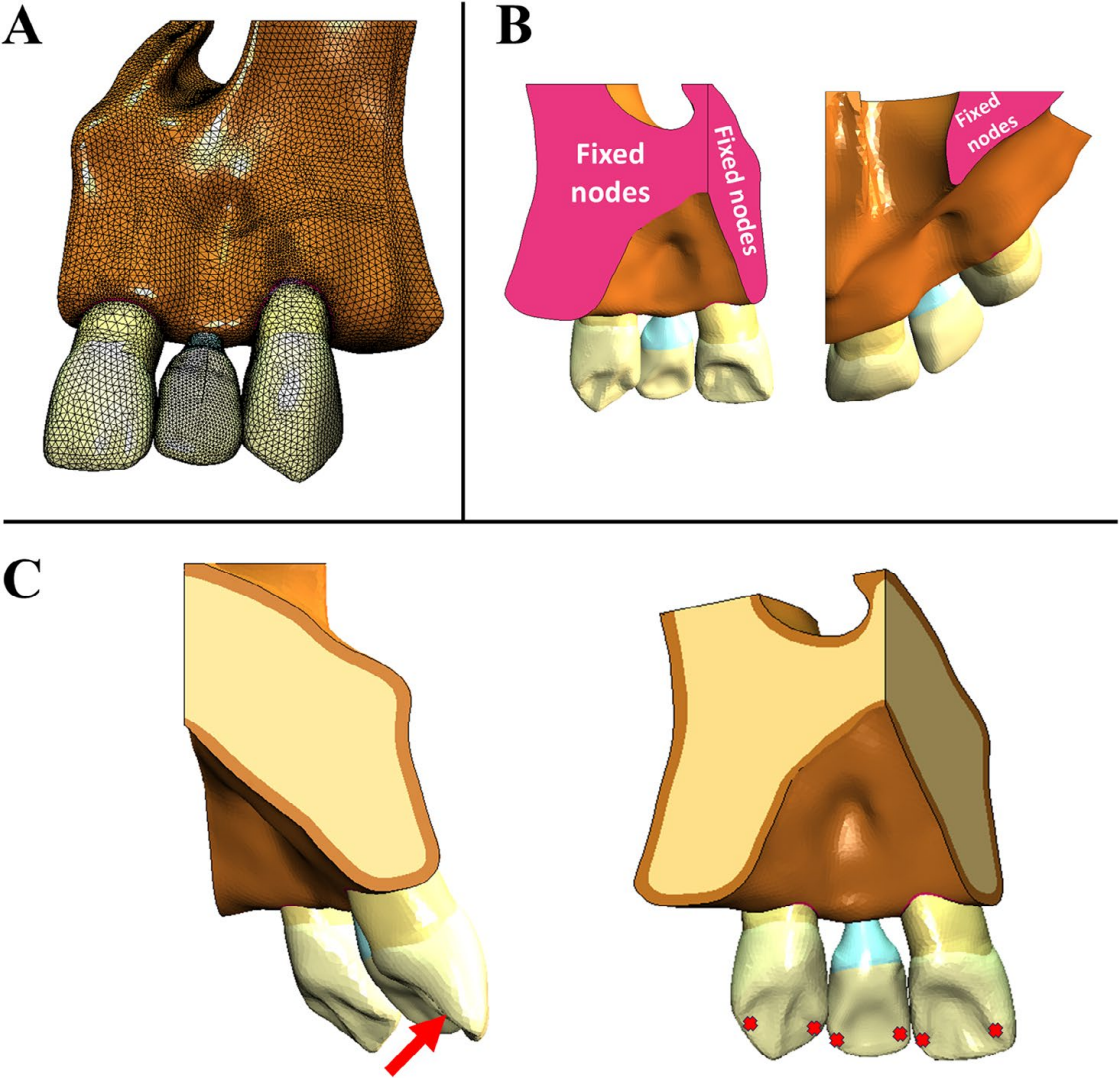
Pre‐processing steps of finite element analysis. (A) Mesh. (B) Boundary conditions in pink color, applied in the nodes of the proximal and superior faces. (C) Oblique loading (45°) of 100 N on each simulated crown.

Loading and boundary conditions were determined using MSC.MarcMentat software (MSC Software, Santa Ana, CA, USA). The boundary conditions were applied by fully constraining the proximal and superior surfaces (corresponding to the maxillary sinus floor) of the simulated maxilla segment in all six degrees of freedom. This condition was implemented to avoid free‐body motion in the simulated models and prevent from an incoherent restriction. All surfaces in contact were modeled as bonded, assuming complete osseointegration between the implant and bone and no sliding among all interfaces in contact. A 100 N oblique load, inclined at 45°, was distributed across two points, with 50 N applied at each location. These load application points were positioned on the mesial and distal margins of the palatal surfaces of the lateral incisor prosthetic crown and the adjacent natural teeth, considering a 2‐mm overbite (Figure 2) This occlusal pattern was based on a distribution of contacts presented by Nelson [42], simulating a physiological clinical condition.

The risks of peri‐implant bone resorption and implant component failure were evaluated. Failure risk assessment for the implant and abutment was conducted based on stress distribution, according to the material behavior. Maximum principal stress criterion was used for zirconia because it is a brittle material, and von Mises stress criterion was used for titanium because it is a ductile material. The failure risk was calculated by the ratio of the maximum stress observed in the implant or abutment to the corresponding critical stress value of the material (titanium or zirconia), according to the following equation:

(1) $$\text{Failure risk}=\frac{\text{Component maximum stress}\;\left(\mathrm{MPa}\right)}{\text{Material critical stress value}\;\left(\mathrm{MPa}\right)}$$

The risk of peri‐implant bone resorption was evaluated based on the strain energy density (SED) distribution in the peri‐implant bone. The pathological SED threshold (Sp) was determined by the conversion of the critical value defined by Frost's Mechanostat [38] (4000 μstrains), which corresponds to the threshold supported by the bone tissue to get into the pathological window. This conversion resulted in an Sp value of 109.6 μJ/mm^3^, as described in a previous study [43]. Additionally, the Peri‐Implant Bone Resorption Risk Index (PIBRri) was calculated by dividing the maximum SED (defined as the mean of the 10 highest SED values observed in the peri‐implant bone elements) by the pathological SED pathological threshold, according to the following equation:

(2) $$\text{PIBRri}=\frac{\text{Peri}-\text{implant bone maximum}\;\mathrm{SED}}{\mathrm{Sp}}$$

The PIBRri value found was attributed to a bone resorption risk according to the following classification [44]: low resorption risk for PIBRri < 0.8; medium resorption risk for 0.8 ≤ PIBRri ≤ 1.0; high resorption risk for PIBRri > 1.0.

### 2.2 Systematic Review of Clinical Studies

This systematic review was performed to assess the clinical outcomes of MLIA rehabilitation using implant‐supported single crowns and to evaluate the consistency between in silico predictions and clinical findings. It was conducted in accordance with the guidelines outlined in Cochrane Oral Health Group's Handbook for Systematic Reviews of Interventions and was conducted following the PICOS strategy:

- P (Population): Studies that evaluated implant‐supported crown as a treatment for MLIA;
- I (Intervention): Dental implants for the management of MLIA;
- C (comparison): Different implant and abutment materials utilized in MLIA rehabilitation;
- O (outcome): Treatment success, esthetic assessment and patient satisfaction;
- S (study design): Clinical studies.

A computerized systematic search was performed in the PubMed, Scopus, and Web of Science databases to identify studies that evaluated implants as a treatment for MLIA, including publications from 2011 onward with the objective to access studies that evaluated dental implants based on the current knowledge. The last search was performed in December 2024. In the initial screening phase, two independent researchers (L.F.R.S. and M.G.R.) reviewed titles and abstracts. Subsequently, full‐text articles were assessed based on the predefined inclusion and exclusion criteria (Table 3) to determine eligibility for inclusion in the present systematic review. The relevant data and main findings from the selected studies were extracted, analyzed and summarized.

**TABLE 3:** Inclusion and exclusion criteria.

Inclusion criteria
Human clinical studies; Clinical studies evaluating implant‐supported crowns for the treatment of MLIA.
Exclusion criteria
Studies that did not address MLIA treatment; Studies that did not use dental implant as a treatment for MLIA; Studies presenting only the orthodontic phase preceding implant placement; In vitro studies, review articles, or author commentaries; Studies involving clef lip and palate cases; Studies focusing on the teeth agenesis in general or other than the MLIA; Studies evaluating implants in regions other than the MLIA site; Use of implants only as anchorage for space closure; Presence of microdontic maxillary lateral incisor instead of agenesis; Use of miniscrew implants as an interim treatment; Studies that did not focus on agenesis treatment or questionnaire for treatment preference; Research not conducted in humans; Studies that did not primarily investigate dental implants; Studies focused on esthetic perception based on crown ratio parameters, photographs or on manipulated images; Insufficient methodological description; Same sample of other publication, but with a shorter follow‐up period; Studies where full‐text was unavailable.

## 3 Results

### 3.1 Finite Element Analysis

**FIGURE 3:**
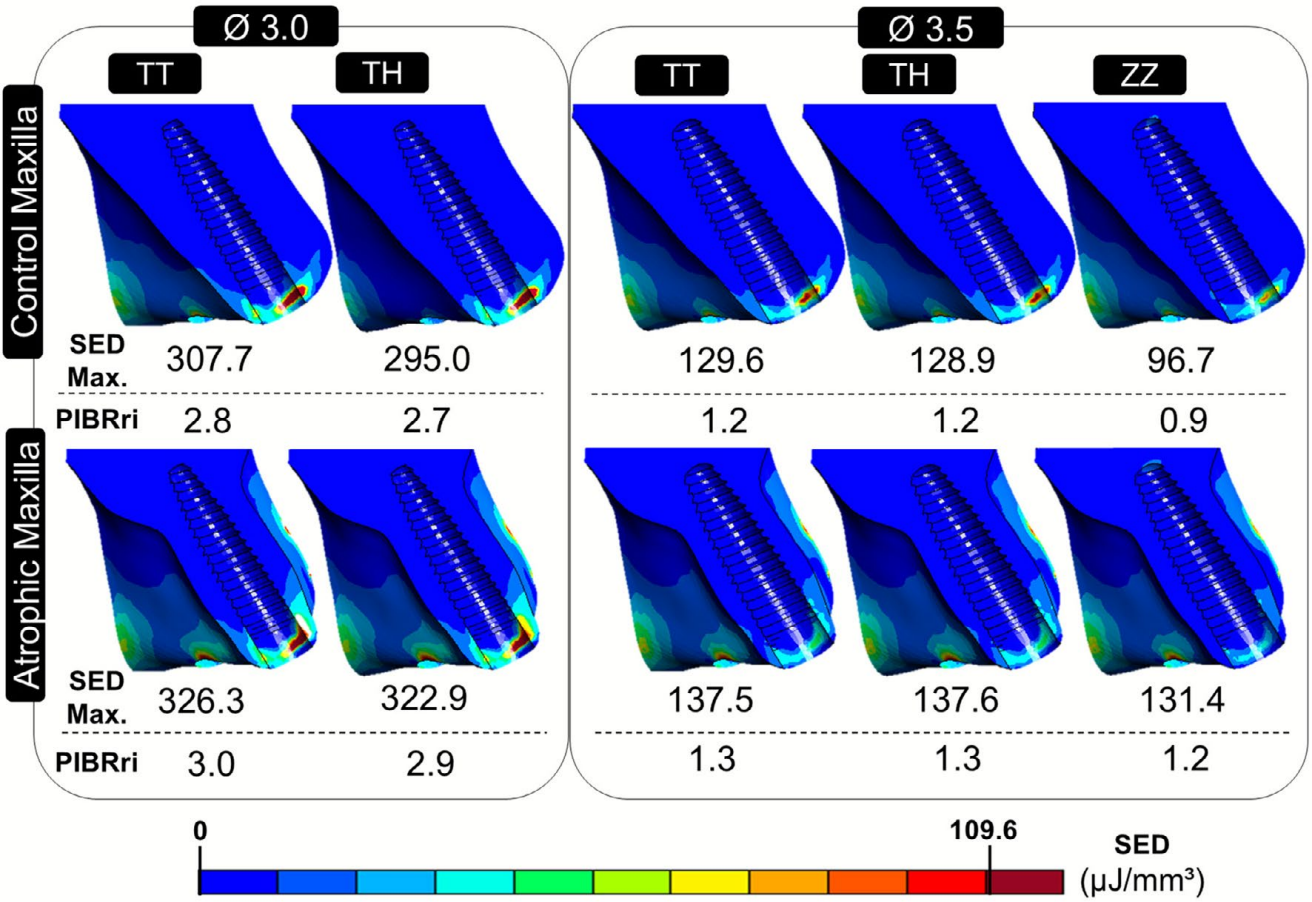
Strain energy density (SED) distribution in peri‐implant bone. Ø, diameter implant in mm; PIBRri, peri‐implant bone resorption risk index; TH, titanium implant and hybrid abutment; TT, titanium implant and abutment; ZZ, zirconia implant and abutment.

Figure 3 presents the strain energy density distribution in the peri‐implant bone across the simulated models. The color scale has been adjusted to emphasize regions with the SED values equal to or exceeding the pathological threshold (109.6 μJ/mm^3^), highlighted in dark red color. Below each model, the corresponding maximum SED and bone resorption risk (PIBRri) are indicated. In all models, the highest SED values are concentrated in the cortical peri‐implant bone adjacent to the implant neck, particularly in the cervical region. Atrophic maxilla models exhibited higher SED values compared to the control models. An increase in implant diameter was associated with reduced SED values. Furthermore, the TT groups presented higher SED values compared to the TH and ZZ groups. All models exceed the pathological SED threshold except the ZZ group in the control maxilla. Moreover, the areas where SED surpasses critical values (in dark red) are limited to a small region of the cervical buccal wall, which is almost invisible for models with 3.5 mm diameter implant of the atrophic maxilla.

**FIGURE 4:**
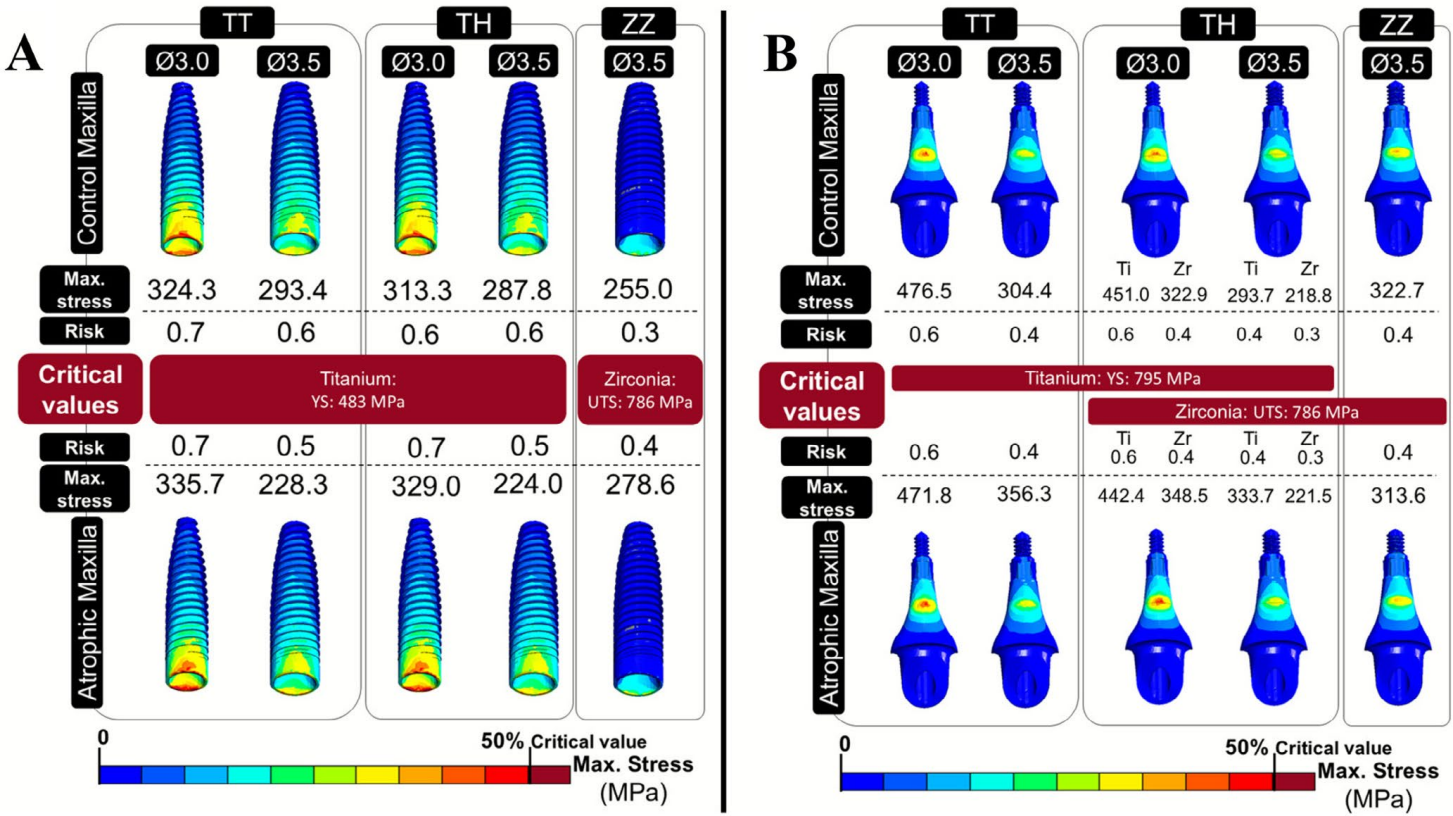
Stress distribution: von Mises for titanium material and maximum principal for zirconia material. (A) Implants. (B) Abutments. The values represent the maximum stress (in MPa) and the failure risk for each component. TH, titanium implant and hybrid abutment; Ti, Ti‐base portion of the hybrid abutment; TT, titanium implant and abutment; Zr, zirconia portion of the hybrid abutment; ZZ, zirconia implant and abutment.

Figure 4A,B show the stress distribution on the implants and abutments, respectively, under varying implant diameters (3.0 and 3.5 mm) and implant and abutment materials (titanium or zirconia). All models are displayed from the buccal view, with the corresponding maximum stress values (in MPa) and failure risk reported beneath each model. For the abutments in the TH group, two stress values are provided: the value on the left corresponds to the Ti‐base component, and the value on the right to the zirconia abutment. The scale was adjusted to 50% of each material's critical failure threshold, based on the respective failure criteria. Regarding implant stress distribution, von Mises stress was predominantly concentrated on the palatal and buccal sides of the cervical portion in titanium implants (TT and TH groups). In contrast, for zirconia implants (ZZ groups), the maximum principal stress was observed only on the palatal side of the same cervical region. Increasing the implant diameter resulted in reduced stress magnitude and more favorable distribution across all implants and abutment materials. Additionally, the use of the zirconia abutments with titanium implants resulted in lower stress values than titanium abutments, for the same titanium implant diameter. However, in the atrophic maxilla, zirconia implant presented higher stress values compared to titanium implants for the same diameter. Regarding abutments, titanium abutments (TT) showed stress concentration on both the buccal and palatal sides, while zirconia abutments (TH and ZZ groups) displayed stress concentration primarily on the palatal side. An increase in implant diameter was associated with reduced stress magnitude for both implant and abutment. Furthermore, for implants with a 3.5 mm diameter, zirconia abutments demonstrated lower stress values compared to titanium abutments. Despite this trend, all implants and abutments in the simulated models presented stress values below the critical value for failure of titanium and zirconia, indicating a low risk of mechanical failure for both titanium and zirconia implant and abutment, under the simulated loading conditions.

### 3.2 Systematic Review of Clinical Studies

**FIGURE 5:**
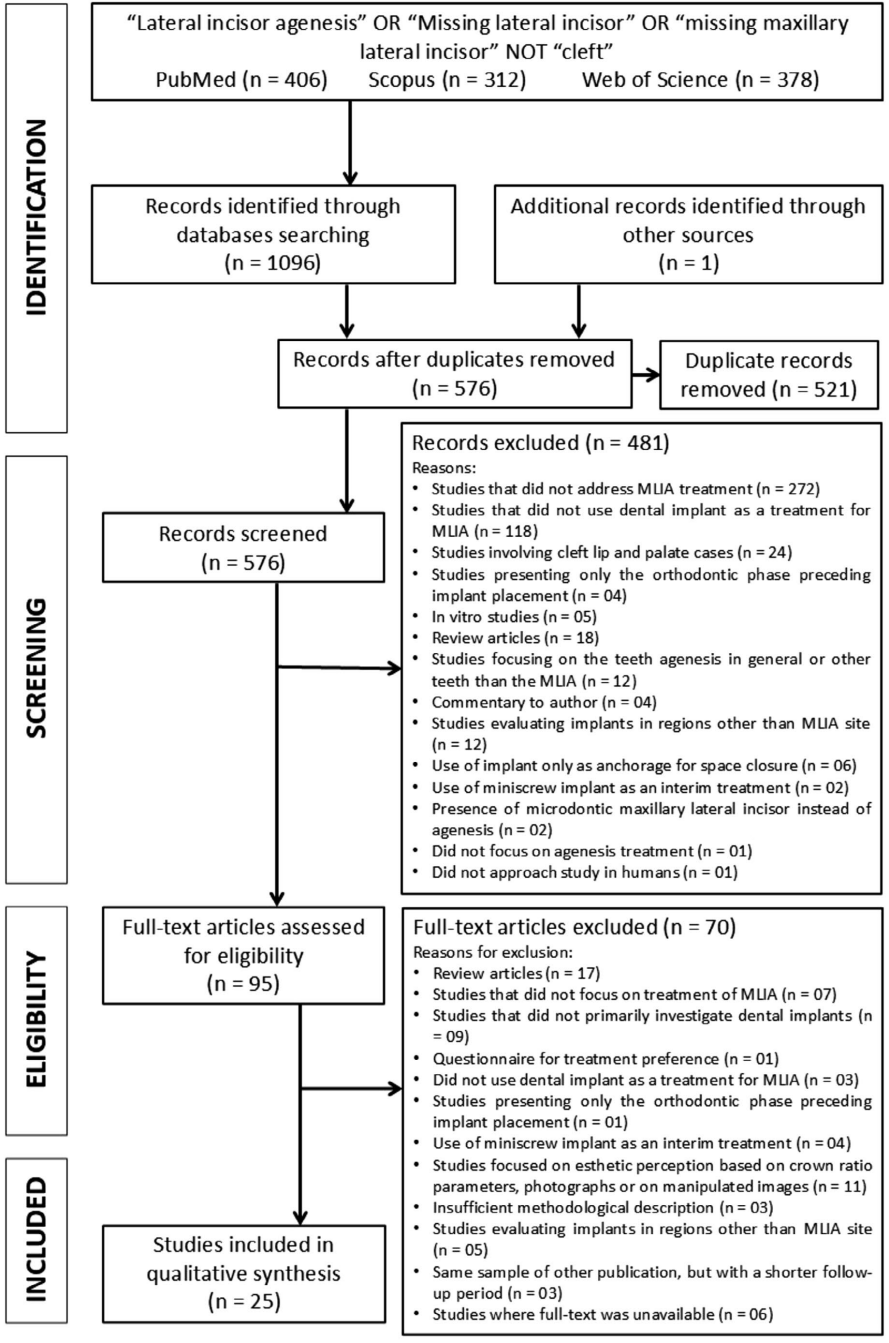
Flow diagram.

The database search identified a total of 1096 articles across the three databases. By using the PRISMA flow diagram, an overview of the article selection process is illustrated in Figure 5. After exclusion of 521 duplicate records, 575 articles remained for further screening. In the initial screening stage, 481 articles were excluded based on titles and abstract review. In the second stage, 94 full‐text articles were evaluated, and 25 studies met the inclusion criteria. A summary of the general data and key findings from these studies is presented in Table 4.

**TABLE 4:** General data and key findings from the included articles.

Study design	Authors/year	Study objective	Main findings	Participants (sex), age, uni or bilateral agenesis	Previous treatment	Follow‐up	Implant dimensions (*n*)	Abutment material
Nonrandomized controlled trial	Marchi et al., 2012 [45]	To assess signs and symptoms of TMD and periodontal status in MLIA cases for different treatments options.	(+) None of the treatments (opening or closure space) were associated with signs and symptoms of temporomandibular joint disorder.	46 (37 F, 9 M), 14–45 years, 19 unilateral, 27 bilateral	Orthodontic	3 years	—	—
	Brazen et al., 2015 [46]	To assess the functional and esthetic outcomes from the professional and patient perspectives in MLIA cases.	(+/−) The outcome of implant‐supported crown in cases of MLIA was very good. Although most patients were satisfied with the esthetics and function, a third of patients desired crown replacement.	36 (19F, 17 M), 16–37 years, 27 unilateral, 9 bilateral	Orthodontic, bone augmentation	5 years	Diameters: 3.3 mm (45) 3.75 mm (9); Lengths: 15 mm (45) 13 mm (9)	Titanium and zirconia
	Mangano et al., 2014 [47]	To evaluate the esthetic outcome of single crown supported by Morse taper connection implants for MLIA.	(+) Good esthetic results, which demonstrates that implant can be used with no esthetic impairment.	20 (11F, 9 M), 19–24 years, unilateral	Orthodontic	3 years	Diameter: from 3.3 up to 4.8 mm	—
	Sorrentino et al., 2024 [48]	To evaluate the 2‐year survival and success rates of implant‐supported zirconia single crowns produced with a digital workflow for the rehabilitation of mono‐ and bilateral MLIA.	(+) The implant‐prosthetic rehabilitation with a full digital workflow proved to be an effective procedure for the treatment of MLIA in the short‐term, with no technical or biological complications.	22 (15F, 7 M), 18–37 years, 14 unilateral, 8 bilateral	Orthodontic	2 years	3.3 × 10 mm	Titanium
	Roccuzzo et al., 2025 [49]	To evaluate the clinical, radiographic and esthetic outcomes of narrow‐diameter implant (NDI) of 2.9 mm in diameter to replace MLIA compared to the same implant type of 3.3 in diameter.	(+) The use of 2.9‐ or 3.3‐mm diameter implants showed comparable favorable mid‐term results in terms of survival rate, bone loss, and esthetic outcomes. Hence, clinicians should rely on the use of such NDIs when replacing.	100 (59F, 41 M), mean age: 21.5 years (range from: 18–31 years), uni or bilateral MLIA	Orthodontic, bone augmentation, bone graft inside socket	3 years	Diameters: 2.9 (50) and 3.3 mm (50)	Titanium
Cohort	Konstantinidis et al., 2016 [50]	To evaluate the stability of peri‐implant soft tissue augmented with the roll flap technique in patients with MLIA.	(+) No prostheses required changes or repair, other than occasional abutment screw loosening. The difference in the PES index between baseline and follow‐up examination was not significant.	17 (10 F, 7 M), mean age: 20.4 years	Soft tissue augmentation	10 years	Diameters: 3.4 (6), 3.8 (9) and 4.5 mm (2)	Titanium and zirconia

	Lacarbonara et al., 2022 [10]	To evaluate the efficacy of mini‐implants in cases of MLIA with severe osseous atrophy.	(+) No patients experienced pain due percussion, all mini‐implants showed no visible vertical and horizontal movements and no alveolar bone resorption.	30 (18F, 11 M), 11 unilateral, 12 bilateral	Orthodontic	10 years	2.7 mm × 13 mm; 3 mm × 13 mm; 3 mm × 11.5 mm	Titanium
Sorrentino et al., 2023 [51]	To evaluate the marginal bone resorption and the peri‐implant soft tissue conditions around Narrow‐Neck ITI implants in rehabilitation of MLIA.	(+) The implant–prosthetic treatment of maxillary lateral incisor agenesis has proved to be a reliable and predictable option from both an esthetic and functional point of view.	72 (49F, 37 M), age range: 19–46 years, uni or bilateral MLIA	—	16 years	Diameter: 3.3 mm; lengths: 10, 12 and 14 mm	Titanium (thick biotype) and zirconia (thin biotype)
Cohort	Hedmo et al., 2024 [52]	To compare the long‐term outcomes of treatments for MLIA over a time span of 10 years to determine whether treatment with implant therapy (IT) and space closure (SC) evolved equally over time.	(+) Among the IT cases, where a difference was found between the cohorts, an improvement was noted. When comparing the cohorts concerning space closure, the color of the crown and overbite had improved over time while the opposite was noted concerning crown length and bleeding on probing.	88 (58 F, 30 M), 19.5–33.7 years, uni and bilateral MLIA	Orthodontic	5 years	—	Titanium
Case report	Avila et al., 2012 [53]	To report a case of bilateral agenesis of MLI in a young patient where the multidisciplinary treatment allowed the resolution of the case with excellence esthetics.	(+) Patient showed satisfactory esthetic and functional results. Implants should be the first choice if there is the possibility of conservative treatment because of the success outcome.	1 (F), 20 years, bilateral	Maxillary expansion, orthodontic, bone augmentation	5 years	3.5 × 13 mm	—
Case report	Collins, 2013 [54]	To assess the use of mini‐implants for a narrow ridge application to restore congenitally missing MLIs.	(+/−) After 2 months of implant placement, examination revealed the lateral implants and composite crown to be slightly mobile due to trauma. After 5 years there were no complaints.	1 (F), 22 years, bilateral	Orthodontic	5 years	1.8 × 13 mm	—

	Jackson and Slavin, 2013 [55]	To demonstrate a multidisciplinary approach for the treatment of MLIA.	(+) The synergy of orthodontics and implant dentistry can solve this condition in an ideal manner. The MLIs and premolars achieved a balanced occlusion (zero contact in centric occlusion, protrusion, and lateral excursion).	1 (M), 43 years, bilateral	Orthodontic, bone graft inside socket, extraction	1 week	3.7 × 13 mm	Zirconia
	Mummidi et al., 2013 [56]	To provide a conservative multidisciplinary approach for the management of bilaterally MLIs.	(+) Space opening made possible to install an implant‐supported crown and get satisfactory esthetic and functional results.	1 (F), 17 years, bilateral	Orthodontic	—	3.5 × 11 mm	—
Oliveira et al., 2013 [57]	To evaluate occlusion relationships in patients with MLIA after space opening and implant insertion.	(+) Adequate canine relationship was achieved on both sides; overbite and overjet were improved.	1 (M), 31 years, bilateral	Orthodontic	3.5 years	Diameter: 3.5 mm	Zirconia
Case report	Martinez‐Rus et al., 2014 [58]	To evaluate esthetic and functional results with a multidisciplinary treatment of a young female patient with MLIA.	(+/−) Fracture of the zirconia abutment after 6 weeks when delivering the definitive crown due to excessive torque to correct the labial emergence of the implant. Substitution of the abutment for a more conservative design to provide adequate wall thickness. Success after 8 months of follow‐up.	1 (F), 20 years, unilateral	Orthodontic, extraction	8 months	3.75 × 13 mm	Zirconia
	Plakwicz, Fudalej and Czochrowska, 2016 [59]	To document the long‐term outcome after transplantation of a developing third molar and the use of a dental implant to substitute for both MLIA in the same patient.	(−) A bluish color was visible through the soft tissues above the implant crown (noticed approximately 2 years after treatment). The total bacteria count was 10 times higher at the implant site than at the transplant and control sites.	1 (F), 19 years, bilateral	Orthodontic, extraction (maxillary second premolars)	9 years	3.5 × 13 mm	—

Alodadi, 2018 [60]	To evaluate the benefit of utilizing three‐dimensional (3D) printed models in managing challenging implant cases in limited arch length for MLIA.	(+) The 3D printing has proved its valuable benefit in treatment planning and executing difficult implant treatment by helping in visualizing the supporting tissues in spatial dimensions and providing accurate measurements and in demonstrating it hands‐on before the surgery.	1 (F), 39 years, bilateral	—	—	3.3 × 12 mm	Zirconia
Case report	Sasaki et al., 2018 [61]	To report the outcomes of a treatment involving computer‐assisted surgery for a bilateral MLIA case and space opening to create sufficient mesiodistal space for implant insertion.	(+) Progress was uneventful, with no prosthetic complications and no bone resorption. Computer‐assisted surgery made possible to correctly the implant placement.	1 (F), 39 years, bilateral	Orthodontic	4 years	3.5 × 10 mm	Zirconia
	Caymaz and Onoral, 2020 [62]	To report an interdisciplinary treatment modality for the patient with MLIA and to explain the way of solution for the biological complication encountered during treatment procedures.	(−) 1 month later, oral examination reinforced with CBCT data indicated aggressive bone resorption around left implant, especially on the buccal wall.	1 (F), 35 years, bilateral	Orthodontic, bone augmentation, soft tissue graft	—	3.5 × 10 mm	Zirconia
	Casula, Gillone and Musu, 2021 [63]	To describe a multidisciplinary approach involving orthodontic treatment to create space for implant placement in a MLIA case.	(+/−) 4 days after interim restoration placement, the keratinized tissue appeared inflamed and was in a more coronal position. At the 24‐month follow‐up visit, no biological or technical complications were noted.	1 (F), 11 years (implant placement: 19 years), bilateral	Orthodontic	24 months	Diameter: 3.3 mm	Titanium
	Stojanović et al., 2021 [64]	To compare two different treatment approaches in cases of unilateral MLIA: space opening and closure.	(+) Some cases need the interdisciplinary team approach for achieving satisfactory results (space opening).	2, 22 and 24 years, unilateral agenesis	Orthodontic	—	Small design implant	—
Case report	Gisotti et al., 2022 [65]	To evaluate the esthetic outcome with two implant‐supported prostheses for a bilateral MLIA case.	(+) An integrated orthodontic, mucogingival, and implantology approach was effective in obtaining a satisfactory implant‐prosthetic rehabilitation from both the functional and esthetic points of view.	1 (F), 24 years, bilateral agenesis	Orthodontic, soft tissue augmentation	1 year	3.3 × 10 mm	Titanium
	Thomé et al., 2022 [66]	To present the outcome of extra‐narrow implants for sites with reduced mesiodistal and buccolingual dimensions.	(+) Use of extra‐narrow Morse Taper implants is a reliable alternative, presenting good outcomes regarding hard and soft‐tissue outcomes.	1 (F), 26 years, bilateral agenesis	—	1 year	2.9 × 10 mm	Titanium
	Zhang et al., 2022 [67]	To described the use of titanium plate and platelet‐rich fibrin (PRF) for an MLIA case.	(+) PRF supplied growth factors and leukocytes for bone and soft tissue regeneration. This procedure reduced the surgical complexity besides demonstrating fewer adverse reactions and outstanding esthetic outcomes.	1 (F), 19 years, unilateral agenesis	Orthodontic, bone graft inside socket, soft tissue augmentation	1 years	3.5 × 13 mm	Titanium
	Iannello et al., 2023 [68]	To demonstrate how a multidisciplinary approach can be implemented to achieve optimal results without complex procedures.	(+) The case was considered completed, and esthetic integration was achieved.	1 (F), 38 years, bilateral MLIA	Orthodontic	N/A	3.5 × 10 mm	Zirconia

Abbreviations: (−), negative outcome for implant therapy; (+), positive outcome for implant therapy; (+/−), positive and negative outcomes for implant therapy; BAPs, bone augmentation procedures; NDIs, narrow diameter implants; PES, pink esthetic score; WES, white esthetic score.

Regarding study designs, 16 studies were case reports (involving one or two patients), five were nonrandomized controlled trials (NCT), with sample sizes ranging from 20 to 100 patients, and four were cohort studies (with sample sizes ranging from 17 to 72 patients). In terms of abutment and implant specifications, 10 studies used zirconia abutment (seven case reports, two cohort studies, and 1 NCT), while none used zirconia implants. The implant diameters varied from 1.8 to 4.8 mm, with the most common diameters being 2.9 and 3.3 mm. Among the 25 studies included, 19 studies reported success outcomes for implant therapy and recommend single implant‐supported crowns in the MLIA rehabilitation, based at the end of the respective follow‐up period [10, 45, 47, 48, 50, 51, 52, 53, 55, 56, 57, 60, 61, 64, 65, 66, 67, 68, 69].

## 4 Discussion

This study integrated FEA with a systematic review to comprehensively assess the biomechanical failure risks associated with implant‐supported crowns in the rehabilitation of MLIA. A patient‐specific three‐dimensional finite element model was modified to consistently simulate an atrophic maxilla with anatomical characteristics typical of an MLIA case, and its outcomes were compared with those of a control maxilla.

Several studies [15, 16, 70, 71] have reported reduced alveolar bone volume at the agenesis site in MLIA patients, leading to the recommendation of the use of narrow‐diameter implants to preserve the limited available bone. In this FEA study, a regular‐diameter implant (3.5 mm) and a narrow‐diameter implant (3.0 mm) were evaluated. While the regular implant failed to maintain the recommended minimum of 1.0 mm of surrounding bone thickness in the atrophic maxilla [72], the narrow implant maintained the recommended bone thickness, but resulted in a higher peri‐implant bone resorption risk (PIBRri) due to mechanical stimuli. Notably, implant diameter exerted a greater influence on the PIBRri than the maxillary bone condition, with similar trends observed in both atrophic and control models.

These findings are consistent with previous FEA studies that reported increased stress concentrations in peri‐implant bone surrounding narrow‐diameter implants (NDI) [19, 73]. This behavior is primarily attributed to the reduced bone‐to‐implant contact area, particularly within the cortical bone, where the highest SED values are concentrated. Despite these biomechanical concerns, clinical studies [74, 75] have demonstrated high survival rates and favorable esthetic outcomes for NDIs, comparable to those achieved with regular‐diameter implants [76]. However, these clinical investigations did not address the specific and challenging clinical context of MLIA.

The present systematic review identified 25 clinical studies that evaluated implant‐supported crowns for the treatment of MLIA, reporting implant diameters ranging from 1.8 to 4.8 mm. Several authors [10, 49, 51, 66] reported favorable mid‐term outcomes for narrow‐diameter implants, including high survival rates, limited marginal bone loss, and satisfactory esthetic results. However, the indication of narrow implants should be limited to cases where insufficient bone volume cannot be corrected through orthodontic space opening or bone augmentation. In scenarios with limited mesiodistal space, orthodontic space opening should be prioritized, as it reduces the risk of peri‐implant bone resorption and promotes better esthetic integration by reestablishing the golden proportion. When the limitation is related to buccopalatal bone thickness, augmentation procedures are recommended.

Among the 25 studies included, 20 reported prior orthodontic space opening, and eight described the use of bone or soft tissue augmentation (Table 4). Soft tissue augmentation is widely recognized as a critical factor for achieving favorable esthetic outcomes, particularly in patients with a thin gingival phenotype [50, 67] where increased soft tissue translucency raises the risk of a grayish shadow caused by the metallic color of the underlying titanium implant, abutment, or even a metal–ceramic crown. To avoid this grayish color, the use of all ceramic crowns associated with ceramic abutments and implants have been proposed [51, 77]. Evidence from a systematic review further suggested that zirconia implants are associated with superior esthetic outcomes in both pink and white indexes, as well as reduced plaque accumulation, which may translate to a lower risk of peri‐implant inflammation [78].

This FEA study compared the failure risk associated with three implant‐abutment material configurations: (1) titanium implant and abutment (TT group), (2) titanium implant with a hybrid abutment, composed of a zirconia portion customized over a titanium base (TH group), and (3) zirconia implant and abutment (ZZ group). All configurations exhibited relatively low failure risks (below 0.7), with the ZZ group showing a slightly lower numerical risk of implant or abutment failure compared to the TT group (Figure 4). However, it is important to emphasize that titanium and zirconia differ fundamentally in their failure mechanisms due to the ductile nature of titanium and the brittle behavior of zirconia. Accordingly, the yield strength (YS) was used to assess the failure risk of titanium, whereas the ultimate tensile strength (UTS) was used for zirconia. From a practical standpoint, if the von Mises stress in the titanium implant reaches its yield strength, the material is expected to fail due to permanent plastic deformation. In contrast, if the maximum principal stress in the zirconia implant reaches its UTS, crack initiation may occur, which can propagate under cyclic loading and potentially lead to catastrophic fracture of the implant.

Zirconia resists crack propagation through a tenacification mechanism involving phase transformation from the tetragonal to the monoclinic phase, which results in a volume expansion of approximately 3%–4% and compression of the failures. However, this mechanism becomes ineffective if the zirconia surrounding the crack is already in the monoclinic phase [79, 80]. Consequently, even with this tenacification mechanism, zirconia's fracture toughness remains relatively low (8 MPa m^1/2^) [36], notably inferior than titanium (range between 60.41 and 85.83 MPa m^1/2^) [81]. Therefore, while FEA results indicated that titanium implants and abutments reached stress values closer to their critical failure (YS values), zirconia implant and abutment remains more susceptible to catastrophic fracture due to subcritical crack propagation.

For abutments, the esthetic benefits of zirconia may justify the increased risk of fracture, since the abutment failure is generally easier to manage clinically. Therefore, hybrid abutments have emerged as a promising solution to balance esthetic outcomes with reduced risk of catastrophic fracture. Linkevicius and Vaitelis [82] conducted a systematic review and concluded that zirconia abutments offer superior esthetic outcomes compared to titanium abutments, particularly in terms of soft tissue appearance measured by the Pink Esthetic Score index. Additionally, zirconia abutments demonstrated comparable performance to titanium abutments with respect to marginal bone loss. Supporting these findings, Watanabe et al. [83] reported that zirconia abutments supported by a titanium base (Ti‐base) exhibit improved biomechanical performance compared to monolithic zirconia abutments.

One study included in this systematic review [58] reported the fracture of a custom angled zirconia abutment 6 weeks after placement, attributed to excessive angulation that compromised the abutment wall thickness. The fractured component was successfully replaced with a less angulated zirconia abutment, showing favorable outcomes after 8 months of follow‐up [58]. Angled abutments are often used in the anterior region to compensate for ridge deficiencies or buccal bone concavities, which are particularly pronounced in MLIA cases. Such anatomical limitations often result in implant positions and angulations that compromise an ideal prosthetic insertion axis [84]. However, angled abutments are associated with increased stress levels on peri‐implant bone and implant components, a higher risk of screw loosening risk [85], and a frequently reliance on cement‐retained crowns, which elevates the risk of peri‐implantitis [86]. For these reasons, their use should be approached with caution when correcting prosthetically unfavorable implant positions. Alternatives strategies, such as angled implants [87] and angled screw channels (ASC) [88], allow for maintaining the advantages of screw‐retained crowns on straight Ti‐base abutments [89]. Although ASC designs have shown promising short‐term outcomes in terms of survival rates and marginal bone stability, current evidence is limited to short follow‐ups (< 3 years), underscoring the need for longer‐term data.

For implants, the risk of subcritical crack propagation remains a critical concern. In vitro studies have confirmed that zirconia implants are more susceptible to fracture compared to titanium implants [90]. In the present study, stress concentration was observed in the cervical portion of the implant, corroborating findings from Sadid‐Zadeh et al. [91], who identified this area as a common fracture initiation site for zirconia implant. Fractures in this portion necessitate additional surgical procedure to remove and replace the implant, which involves a more complex treatment. Long‐term clinical studies are still required to validate the longevity and performance of zirconia implants and to determine whether their success rates can be comparable to those of titanium implants over extended periods of function [92, 93, 94]. To date, available evidence on zirconia implants survival rate is primarily based on short‐term follow‐ups, not exceeding 5 years [78, 95]. Therefore, it is not surprising that none of the studies included in the systematic review employed zirconia implants.

Among the 16 case reports included in this systematic review, 14 documented successful outcomes, with no surgical, biological or prosthetic complications during the follow‐up [53, 55, 56, 57, 58, 59, 61, 63, 64, 65, 66, 67, 68]. Similarly, three clinical trials reported 100% implant survival rates [46, 47, 48], while Roccuzzo et al. [49] reported a survival rate of 100% for 2.9 mm implant diameter and 98% for 3.3 mm implants due to a single early implant failure. Success rates were also high: Mangano et al. [47] reported no complications, whereas Branzén et al. [46] observed minor esthetic and technical complications, including crown fracture, incomplete papilla fill, and patients' dissatisfaction with crown appearance. Roccuzzo et al. [49] reported screw loosening and retention loss in both diameter groups, along with soft tissue discoloration in 13% of the group Ø2.9 mm and 2.4% of the Ø3.3 mm group. One study found no significant differences in temporomandibular joint (TMJ) disorders between implant therapy and space closure approaches for MLIA [45].

Along the four cohort studies included in this systematic review, follow‐up ranged from 5 and 16 years. Lacarbonara et al. [10] reported 100% survival and no bone resorption in narrow titanium implants (2.7 and 3.0 mm) over 10 years. Hedmo et al. [52] compared the outcomes of an early cohort (patients treated between 2001 and 2008) with a late cohort (patients treated between 2011 and 2018) and found improved outcomes over time for both implant therapy and space closure. However, in the space closure group, the bleeding on probing index got worse comparing the early cohort with the late cohort. Konstantinidis et al. [50] and Sorrentino et al. [51] compared titanium and zirconia abutments over 10 and 16 years, respectively. Konstantinidis et al. reported 100% of survival rate with no signs of peri‐implantitis in either group. Sorrentino et al. observed a slightly lower survival rate (93.3% for titanium abutments and 95.7% for zirconia abutments), with complications including esthetic failure, ceramic chipping, loss of retention, mucositis, one case of severe peri‐implantitis in the titanium group, and two cases of mild peri‐implantitis in the zirconia group. In general, the evidence supports the use of single implant‐supported for the rehabilitation of MLIA, demonstrating high survival rates and favorable esthetic and functional outcomes over time.

Overall, the studies included in this systematic review report promising outcomes for implant‐supported rehabilitation in cases of MLIA, suggesting a potential shift in clinical decision‐making toward implant‐supported solutions as a viable and esthetically favorable treatment option. These findings highlight the importance of a multidisciplinary approach when selecting implant‐supported crowns as a treatment modality for MLIA, integrating orthodontists, periodontists, implantologists, and prosthodontists to optimize treatment planning and achieve predictable and successful outcomes [64, 65]. The process involves several essential steps, including orthodontic space creation, soft and hard tissue augmentation to establish a stable foundation, and the use of narrow‐diameter implants when indicated. Additionally, a provisional crown is used to establish the proper emergence profile, with the final prosthetic design being critical for achieving an optimal esthetic result [96]. Furthermore, clinicians must be well‐informed about potential complications and have the necessary skills and treatment options to manage them effectively [97, 98].

Still, some limitations of the present study must be addressed. This FEA study was conducted under idealized post‐osseointegration conditions, assuming perfect union between model components. Multiple clinical variables, such as patient systemic health, oral hygiene, surgical procedure, and implant primary stability, also play critical role in peri‐implant bone resorption and were not accounted for. Furthermore, the same implant geometry was considered for both titanium and zirconia implants, despite variations in commercially available zirconia implant designs that may influence stress distribution and failure risk. Factors such as manufacturing‐induced microcracks, which can significantly affect zirconia performance were also not considered in the simulation. Lastly, while this systematic review included clinical studies on implant‐supported crowns for MLIA, none specifically evaluated zirconia implants. Current evidence for zirconia implants in the anterior region remains limited to short‐term follow‐ups, highlighting the need for long‐term clinical studies to validate the longevity and performance of zirconia implants in MLIA cases.

## 5 Conclusions

By integrating the evidence from both systematic review and the finite element analysis, it was concluded that:

- The implant–prosthetic rehabilitation of MLIA has demonstrated high reliability and predictability in both esthetic and functional outcomes, especially when performed through a multidisciplinary approach involving orthodontics, periodontics, implantology, and prosthodontists.
- The use of narrow implants is indicated to cases where insufficient bone volume cannot be corrected through orthodontic space opening or bone augmentation.
- The titanium implant with a hybrid abutment, composed of a zirconia portion customized over a ti‐base, was shown as a reliable alternative, offering favorable biomechanical behavior while maintaining the esthetic advantages of zirconia in the transmucosal zone.
- Long‐term studies are still necessary to validate the longevity and performance of zirconia implants in MLIA.

## Funding

This work was supported by Fundação de Amparo à Pesquisa do Estado de São Paulo (2023/07312‐0).

## Conflicts of Interest

The authors declare no conflicts of interest.

## Data Availability

The data that support the findings of this study are available on request from the corresponding author. The data are not publicly available due to privacy or ethical restrictions.
